# *inserexs*: reflection choice software for resonant elastic X-ray scattering

**DOI:** 10.1107/S1600576723002212

**Published:** 2023-04-13

**Authors:** Antonio Peña Corredor, Nathalie Viart, Christophe Lefevre

**Affiliations:** a Université de Strasbourg, CNRS, IPCMS, UMR 7504, 23 rue du Loess, Strasbourg 67200, France; Ecole National Supérieure des Mines, Saint-Etienne, France

**Keywords:** *inserexs*, resonant elastic X-ray scattering, REXS, EXAFS, atomic positions, atomic occupancies, thin films

## Abstract

This paper presents *inserexs*, an open-source computer program that aims to pre-evaluate the different reflections for resonant elastic X-ray scattering (REXS) diffraction experiments. REXS is an extremely versatile technique that can provide positional and occupational information about the atoms present in a crystal. *inserexs* was conceived to help REXS experimentalists know beforehand which reflections to choose to determine a parameter of interest. Prior work has already proven this to be useful in the determination of atomic positions in oxide thin films. *inserexs* allows generalization to any given system and aims to popularize resonant diffraction as an alternative technique to improve the resolution of crystalline structures.

## Abstract 2

*inserexs* is an open-source program that allows resonant elastic X-ray scattering users to choose the best conditions to determine any parameter of interest (atomic position, occupancy *etc*.) before an experiment is carried out.

## 1. Introduction

Precise location of atoms is crucial to explain the properties of many functional materials. In some ferromagnetic materials, the magnetic ordering can only be explained via the atomic positioning of the crystal cations (Giovannetti *et al.*, 2011 ▸). In some ferroelectric oxides, knowing where oxygen vacancies are can be the key to understanding the phenomena behind the polar behaviour of the material (Zhou *et al.*, 2019 ▸). Equally, in oxides with an orbitally ordered state, knowing the position of oxygen atoms allows understanding of the lattice deformations which lead to the orbital order inside the material (Ishibashi *et al.*, 2017 ▸).

In bulk materials, X-ray diffraction (XRD) or neutron diffraction can be used to locate the different atoms that compose the crystal (Woińska *et al.*, 2016 ▸; Shahzad *et al.*, 2011 ▸). But these conventional diffraction methods struggle to provide the whole picture of a crystal when it is presented as a strongly orientated system (*e.g.* thin films) or when there is little matter to probe (*e.g.* nanoparticles). More adapted methods might raise some other issues. For instance, precession electron diffraction (Rotella *et al.*, 2015 ▸) allows the crystallographic structures of thin films to be solved, but requires a tedious and destructive sample preparation process.

**Figure 1:**
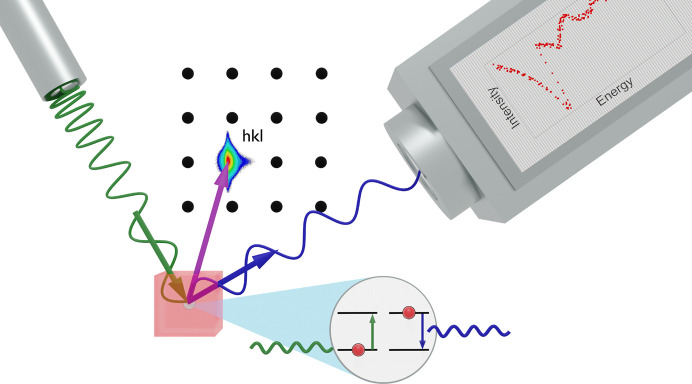
Schematic of a REXS setup and experiment. Green – incident beam; blue – diffracted beam. The X-ray source, target material and X-ray detector are oriented so that the diffraction condition is respected for the desired reflection (*hkl*).

As an alternative, resonant elastic X-ray scattering (REXS) has shown success in locating atoms in thin films in a non-destructive and straightforward manner. REXS, also known as anomalous diffraction, combines X-ray absorption spectroscopy (XAS) and XRD in a single experiment (Hodeau *et al.*, 2001 ▸). XAS provides information about the electronic states involved in the crystal ordering, whereas XRD provides information about the spatial order. In a REXS experiment, an incident photon (of varying energy) excites a bound core electron into an unoccupied state, close to the Fermi level, while the orientation of the material respects the diffraction condition – the scattering vector is aimed at a specific reciprocal lattice point (RLP). The electronic transition from a ground to an excited state strongly depends on the electronic structure of the valence shell. The electron falls back into its core ground level, re-emitting a photon of the same energy as the incoming photon (Fink *et al.*, 2013 ▸). Since the transition takes place at a specific edge of a specific atom (among those present in the crystal), REXS is both element and orbital sensitive (Vettier, 2012 ▸). This process, as well as a visual scheme of the experimental setup, are represented in Fig. 1 ▸.

By studying the X-ray absorption near-edge structure (XANES; 50 eV around the absorption edge), a technique also known as diffraction anomalous near-edge structure (DANES), information about the oxidation state of the absorbing atoms can be retrieved (Kawaguchi *et al.*, 2014 ▸). Beyond this edge, by scanning the extended X-ray absorption fine structure (EXAFS; 50–1000 eV above the edge), also known as extended diffraction absorption fine structure (EDAFS), we can obtain information about the chemical environment (Poineau *et al.*, 2019 ▸). In thin films, DANES has already been used to study cation oxidation states and their occupation factors (Lefevre *et al.*, 2017 ▸), and we have recently shown that EDAFS can be successfully used to indirectly locate the ligands of the cation (Peña Corredor *et al.*, 2022 ▸).

In contrast to conventional XRD, REXS exploits the energy dependence of the dispersive terms [*f*′(*E*) and *f*′′(*E*)] of the atomic scattering factor [ 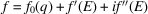 ] by measuring the intensity as a function of the energy at a specific RLP. These anomalous terms or the crystal structure factor, however, are not the same for all the crystal reflections. As a result, to determine a certain parameter (an atom position, a site occupation), all the RLPs will not be equally useful to provide the desired information.

The symmetry conditions for an RLP to be active in diffraction experiments (allowed reflections) are determined by the crystal symmetry and the different atomic scattering factors. A reflection is ‘allowed’ when the interference between the scattered waves is not destructive. However, the general symmetry conditions are only applicable when the equivalent positions are occupied by atoms with the same atomic scattering factor. In fact, the interatomic interactions may break the atoms’ sphericity, and the scattering factors of equivalent atoms could be different. In this case, ‘forbidden’ reflections can have a non-zero structure factor. This can happen naturally via thermal atomic vibrations or point-defects in the crystal (Dmitrienko & Ovchinnikova, 2002 ▸). Moreover, if the X-ray susceptibility of the crystal is anisotropic, the forbidden reflections can be observed by characterizing the near-edge absorption spectrum, thus giving a non-zero intensity in REXS experiments (Dmitrienko, 1983 ▸). Conversely, the non-equality of the scattering factors of crystallographically equivalent atoms can cancel out the structure factor for conventionally allowed reflections. The suppression of an otherwise allowed reflection makes that reflection highly sensitive to any atomic displacement (Richter *et al.*, 2018 ▸). In conclusion, the choice of a convenient RLP is crucial for the determination of a parameter of interest.

In an ideal case, in order to solve a crystallographic structure, one could simply measure the REXS spectra at all non-null reflections. However, in real experiments, the acquisition of DANES/EDAFS spectra at all RLPs is not possible because of the measurement times that would be required. Access to instruments that can facilitate such experiments is costly and infrequent and beam times are limited. Prior to the measurement, an experimentalist should know whether REXS experiments are suitable for their aim and decide which are the most appropriate reflections.

Here we present our application *inserexs* (intensity and sensitivity comparator for REXS), conceived to help REXS users decide which reflections should be explored in order to determine a certain parameter of interest.

**Figure 2:**
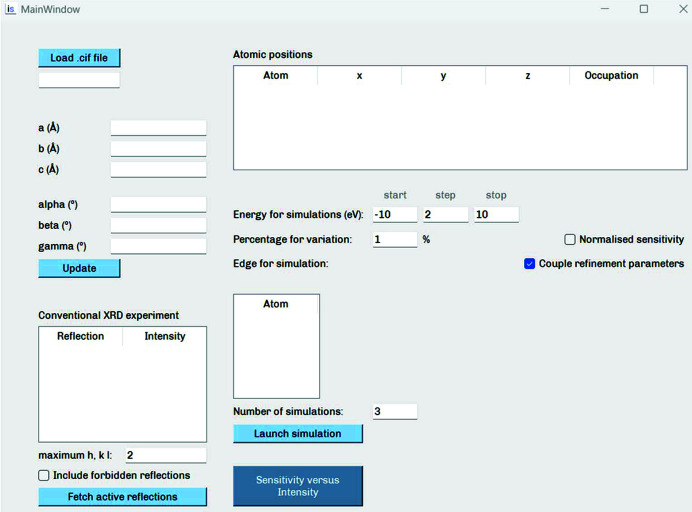
User interface for *inserexs*.

The framework was written in Python and consists of several modules that communicate between each other, as detailed later. Implemented multithreading capability allows the simultaneous operation of the several modules that compose the general program. Simulation of the spectra is performed with the *ab initio* software *FDMNES* (Bunău & Joly, 2009 ▸), which uses time-dependant differential functional theory (TD-DFT) with the Hubbard correction (LDA+U) for the generation of spectra. The user interacts with *inserexs* through an intuitive graphical user interface (GUI), shown in Fig. 2 ▸, from which the user can load all the crystallographic data, specify the desired parameters from REXS and visualize the program output. The option of inserting the data without the GUI is also available. *inserexs* has been tested on Windows 10 and Ubuntu 20.040 LTS. At its current stage, *inserexs* has already accomplished the goal for which it has been designed: facilitating the choice of reflection for the determination of an atomic position or occupancy, before any real experiment takes place. Future releases will expand its current functionalities. An expanding user manual has been included in the software distribution.

## 2. Feature overview

### 2.1. Reflection choice: intensity and sensitivity

Judging the adequacy of each reflection to determine a certain parameter is the main objective of *inserexs*. This ‘usefulness’ or adequacy will depend on two factors. First, the more intense a reflection, the better the signal-to-noise ratio for the measurement, and the better the spectrum fitting and the refinement. Therefore, the intensity of the reflection will be the first parameter to consider. For this intensity value, *inserexs* calculates the crystal structure factor 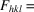 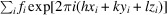 and considers the intensity to be 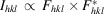 . This allows the program to know which reflections are allowed (non-zero intensity). For such a purpose, *inserexs* generates all the atomic positions (from the symmetry operations and the positional information in the .cif file) and calculates *F_hkl_*, considering the atomic scattering factor (*f_i_*) of each atom. By default, *inserexs* considers a reflection to be active when its intensity is larger than 0.1% of the intensity of the most intense reflection. This value can be manually changed in the intensity module.

For quick simulations, *inserexs* makes a rough approximation by considering the atomic scattering factor to be the number of electrons of the atom. However, on request (by selecting ‘Include forbidden reflections’ in the GUI), *inserexs* can precisely calculate the *f_i_* of each atom using the numerical tables of anomalous scattering factors calculated by the Cromer and Liberman method (Sasaki, 1989 ▸). For this last method to work, the file Sasaki_anomalous (distributed alongside *inserexs*) must be present in the directory where *inserexs* is executed. If the atom whose edge will be used for the simulations is indicated, the edge energy of that atom is used for the calculation of the atomic scattering factor. Otherwise, the atomic scattering factors are calculated for an energy of 10 keV, *i.e.* the order of magnitude of most cation *K* edges (not recommended). Independently of choosing whether or not to consider the forbidden reflections, the plotted intensity will correspond to the one generated by *FDMNES*, for which each atomic scattering factor is individually calculated again.

**Figure 3:**
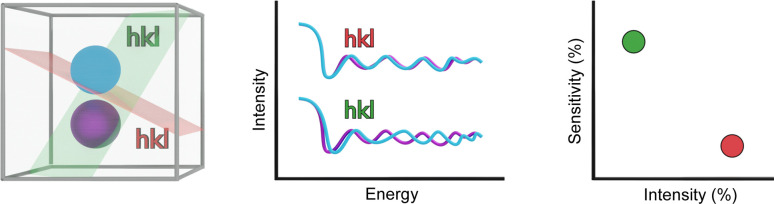
Left: scheme of two possibilities for an atomic position (blue and purple) and two possible *hkl* reflections (red and green) at which REXS experiments can be carried out. Middle: comparison of REXS spectra acquired at the two reflections. The red *hkl* is shown to be more intense, whereas the green *hkl* is more sensitive to changes in the atomic position. Right: comparison in the sensitivity versus intensity plot for the two reflections.

Secondly, the REXS spectrum must be sensitive to the parameter of interest: if this parameter changes, the spectrum acquired at a certain reflection must also change, and the spectrum modification must allow detection of the parameter variation. For this, one can define a second parameter called ‘sensitivity’ which considers how much spectra are altered when the desired parameter changes. These two parameters and the way in which *inserexs* uses them to evaluate each reflection are schematized in Fig. 3 ▸.

As shown in equation (1), the sensitivity calculation for a specific RLP compares the simulated intensity [*I*_TS(*x*)_] for a specific set of parameters (*x*) and at a specific energy *E* to the average simulated intensity at this specific energy, for all sets of parameters, *I*_av_(*E*):

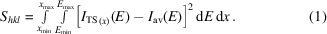

The sensitivity is proportional to the intensity since it sums the absolute spectral variation. A ‘normalized’ sensitivity can also be proposed, which instead takes into account the relative spectral variation, as shown in equation (2):

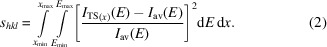

The normalized sensitivity, however, is overestimated for reflections with a very low intensity and, as a result, the ‘absolute’ sensitivity will be the default option for *inserexs*. The normalized sensitivity is also always calculated, and switching from one to the other can be done on request by checking that option on the GUI.

Prior assessment of the intensity and sensitivity of reflections has already proved useful for indicating which reflections to choose and to verify that the REXS experiments were worth performing, when we attempted to determine the position of oxygen atoms in thin films (Peña Corredor *et al.*, 2022 ▸).

### 2.2. Code composition and module interaction

**Figure 4:**
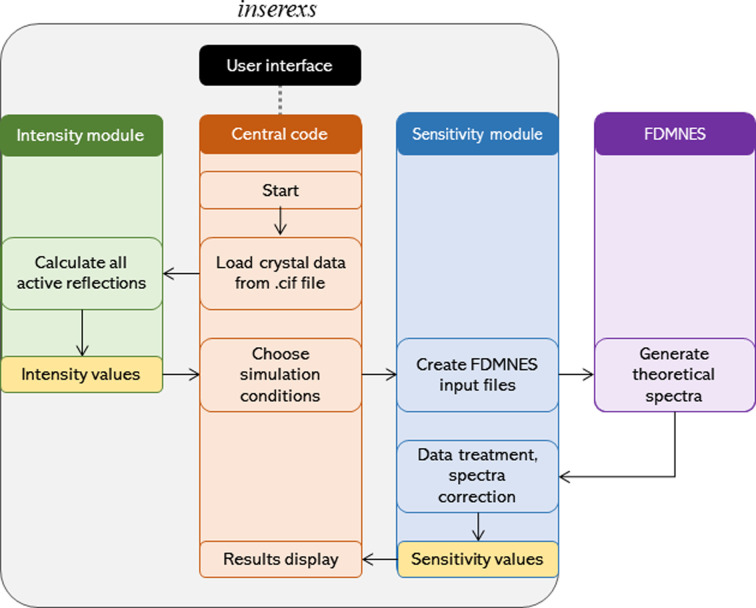
Data flowchart for the calculation of the intensity and sensitivity values from an input file, showing the interaction between the program modules and between the modules and *FDMNES*.

The program is composed of three main modules which interact with the outer program *FDMNES* as shown in Fig. 4 ▸. The first is the central module, whose function is to connect the rest of the modules and provide a backbone to the whole application. It is also directly linked to the GUI, where the input takes place. It is further responsible for data assembly and final display. The other two modules are in charge of obtaining the intensity and sensitivity values, respectively.

In the GUI, the user can load the crystal data as a .cif file. Alongside the *inserexs* main code, a ‘cif builder’ tool has been distributed for cases where a .cif file is not available. The .cif file should follow the conventions indicated in *International Tables of Crystallography* (Bernstein *et al.*, 2016 ▸). The code attempts to extract the different crystal parameters from the CIF, and these can be subsequently modified and updated directly from the GUI. Several .cif files have been successfully tested, each one with different writing styles and amounts of information. However, some lines will be mandatory for the correct functioning of *inserexs*. The complete list can be found in the user guide.

All the CIF information is sent to the intensity module, which uses the crystal information to obtain the intensity of the non-null reflections. For this purpose, the program generates all the atomic positions from the .cif file’s atomic positions and symmetry operations. Then, redundant positions are removed and each unique position is used to calculate the structure factor for each reflection. By using the lattice parameters, we can verify whether the reflections are equivalent, and output those which are not. From the GUI the user can choose to either neglect all forbidden reflections or take them into account (despite not being observed in conventional XRD experiments they can sometimes be measured in REXS). A maximum value for the *h*, *k* and *l* values must be entered (2 by default). In order to calculate all the possible reflections, a large number should be introduced. If the forbidden reflections are considered the calculation time significantly increases. For the intensity calculation, the module calculates the Thomson component of the atomic scattering factor, as well as its anomalous components (Brown *et al.*, 2006 ▸). Then, it calculates the structure factor, considering both the atomic scattering factors and the atomic positions. The output intensity has been simplified as the square of the structure factor.

The code considers a general triclinic case when angle-dependent calculations are needed for the atomic scattering factor. The set of intensities for each reflection can be retrieved from all these steps, although similar results are produced (and faster) using the intensities given by *FDMNES*. The code requires the user to choose from the GUI which parameter will vary, how much it will vary, the energy range (start, stop and step) and the edge on which the simulation will take place. The user can also choose if all the parameters will vary simultaneously or if they should vary in an unrelated manner. All this information, alongside the list of active reflections, is sent to the sensitivity module so that valid *FDMNES* input files are generated. *FDMNES* is then run and generates the simulations. A cluster radius of 5 Å was chosen after multiple trials (a good compromise between exact results and reasonable calculation time). The Green formalism is used for the calculations.

If the chosen edge or the chosen parameter does not respect the crystal structure, an error arises at this point. If the process has been successful, the sensitivity module loads the *FDMNES* output data, treats them and calculates the sensitivity as previously defined. Finally, a sensitivity versus intensity plot is shown directly in the GUI. The plot is also automatically saved in the folder where the .cif file was found.

### 2.3. Output showcase

Three examples are given in this section. They are analogous to the study we carried out on FeV_2_O_4_ (Peña Corredor *et al.*, 2022 ▸), in which both the experimental and the theoretical spectra were shown. In the structures presented hereafter, the studied parameter directly affects the material properties, and the best reflections to study such parameters are plotted in a sensitivity versus intensity plot.

**Figure 5:**
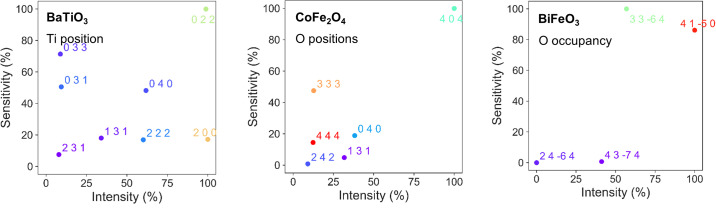
Results for the different systems (and varying parameters): BaTiO_3_ (Ti position), CoFe_2_O_4_ (O positions) and BiFeO_3_ (O occupancy).

All the parameters have been modified by 1%. The results are shown in Fig. 5 ▸. An energy step of 2 eV was chosen for the simulations, for percentual variation of the indicated parameters of 1%. A maximum value of 4 for *h*, *k* and *l* was chosen. The first example corresponds to a DANES experiment and aims to locate the cation which is directly explored, while the two others correspond to EDAFS experiments in which cations are probed to indirectly obtain the positions or the occupancies of the oxygen atoms.

Firstly, we consider the ferroelectric oxide BaTiO_3_ (*P*4*mm*), whose polar behaviour can be attributed to the displacement of the Ti^4+^ cation in the perovskite structure (Barrett *et al.*, 2010 ▸; Kwei *et al.*, 1993 ▸) along the *z* direction. The XANES region of Ti (−50, +50 eV around the edge), which is sensitive to the cation position, has been chosen. The 022 reflection is shown to be the most sensitive to the Ti position and also the most intense, whereas the 200 reflection is just as intense but not as sensitive. Another suitable reflection would be 040, being both sensitive and intense; or 033, also sensitive to the parameter under study.

The second system is the spinel oxide CoFe_2_O_4_ (*Fd* 3 *m*), a hard ferromagnetic material with good electromagnetic performance (Maaz *et al.*, 2007 ▸) and catalytic properties (Cao & Zuo, 2020 ▸). It crystallizes in a cubic spinel structure, with oxygen atoms located in the 32*e* position, which depends on the *x* parameter (*x*, *x*, *x*) (Wyckoff, 1931 ▸). To determine this position, the EXAFS part of one of the cations could be chosen (*e.g.* +20, +120 eV). For this material, and in contrast to the previously studied FeV_2_O_4_ (Peña Corredor *et al.*, 2022 ▸), the 404 reflection is the most intense and the most sensitive to the oxygen positions. The difference might arise from the difference in Fe—O polyhedral configuration: tetrahedra in FeV_2_O_4_ versus octahedra in CoFe_2_O_4_, leading to distinct EXAFS signatures when the oxygen positions vary.

The third crystal studied is the ferroelectric BiFeO_3_ (*R*3*c*:H), whose electrical polarization and switching dynamics are highly dependent on the oxygen vacancies (Noguchi *et al.*, 2019 ▸; Sosnowska *et al.*, 2013 ▸). In order to determine the occupation of the oxygen site, the same conditions for the EXAFS domain have been chosen at the Fe edge. The 33 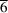 4 reflection is clearly the most sensitive to the occupation of the oxygen atoms and is also intense enough to be considered. The 41 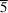 0 reflection is the most intense and also possesses significant sensitivity. In this example these two reflections are so intense and sensitive with respect to the occupation factor of the oxygen that the remaining reflections have almost zero relative intensities and sensitivities.

## 3. Conclusions

We have shown how the *inserexs* code can evaluate the active reflections of a system for a REXS experiment, considering both their intensity and how sensitive they are with respect to the studied parameter. The program has been demonstrated on three completely different materials, for three different purposes and using different simulation conditions. From a user-friendly GUI interaction, the program uses a .cif file to send the crystallographic information to its several modules, from which the intensity and sensitivity values are obtained. This program will help any potential REXS user in deciding which reflections to choose before any real experiment takes place.

## 4. Distribution

*inserexs* has an open-source code which is protected under a BSD licence at the Agence de Protéction de Programmes (French Agency for software protection; deposition No. IDDNFR001490010000SP202200031325). It is free to use or modify with the appropriate authorship recognition and can be found at https://github.com/antpeacor/inserexs.
